# Tooth Abnormalities In Individuals With Unilateral Alveolar Clefts: A Comparison Between Sides Using Cone-Beam Computed Tomography

**DOI:** 10.4317/jced.54043

**Published:** 2017-10-01

**Authors:** Bruno-Torres Bezerra, John-Nadson-Andrade Pinho, Luiz-Carlos-Ferreira da Silva

**Affiliations:** 1 DDS, MSc.) Professor, Department of Stomatology, School of Dentistry, Tiradentes University, Aracaju, Brazil; 2 DDS, Dental surgeon, Private Practice, Aracaju, Brazil; 3 DDS, MSc, PhD, Professor, Department of Oral Surgery, School of Dentistry, Federal University of Sergipe, Aracaju, Brazil

## Abstract

**Background:**

Tooth abnormalities are most often present in individuals with oral clefts than general population, and lead to a long-term impact on facial anatomy and self-esteem. The purpose of this study was to compare the proportion of dental anomalies between the cleft side and non-cleft side in individuals with non-syndromic unilateral alveolar clefts (AC).

**Material and Methods:**

Twenty cone beam computed tomography (CBCT) scans were converted into three-dimensional (3D) virtual models. The dental anomalies considered were: tooth agenesis; supernumerary teeth; giroversion; and microdontia. Statistical analysis was performed using the McNemar’s test and Fisher’s exact test (*p*<0.05).

**Results:**

Statistically significant differences were not found either between the prevalence of individuals with dental abnormalities on the non-cleft side and the sides of the AC (*p* = 1.00), or sex (*p* = 0.36). Tooth agenesis was the most prevalent dental anomaly (55.6%). On the cleft side the lateral incisor was tooth most involved by dental anomalies; and the second premolar was the most affected on the non-cleft side.

**Conclusions:**

This study showed a high frequency of dental anomalies in the cleft individuals and indicated that the side of AC and sex do not interfere in the proportion of dental anomalies on non-cleft side.

** Key words:**Cone beam computed tomography, Hypodontia, Tooth abnormalities.

## Introduction

The development of facial structures and oral cavity follows a complex sequence of events involving the coordination for cell migration, growth, differentiation, and apoptosis. Disturbances in these events between the fifth and tenth week of fetal life may affect the development or embryonic processes fusion, resulting in the formation of orofacial clefts (1).

The individual with cleft lip and palate requires a multidisciplinary specialized care from birth to adulthood to reduce the difficulties imposed by clefts such as social integration, feeding, breathing, hearing, speech, appearance, and dentition. Dental anomalies represent one of the abnormalities that accompany the cleft individual, leading to a long-term impact on facial anatomy and self-esteem (2).

It has long been known that cleft patients present dental anomalies more often than general population (3-5), as well as the frequency of anomalies appears to increase with the severity of the cleft (5,6). Anomalies of number, shape, size, and position may be found in this group of individuals on the cleft or non-cleft side. Recently, some studies have suggested that dental anomalies can be clinical markers and define more specific sub-phenotypes of oral clefts (7,8).

Radiographic examination helps to identify the numerical, morphological or eruptive anomalies on teeth. Increasingly, cone beam computed tomography (CBCT) has become a widely known diagnostic tool for oral and maxillofacial region (9,10). Three-dimensional (3D) images obtained from CBCT exhibit high level of observable details from different angles in contrast to conventional two-dimensional radiographs (2D). Although panoramic radiographs have good diagnostic value for dentofacial structures, some of their limitations including distortion, magnification, superimposition of craniofacial structures, positional problems and low reproducibility of details may adversely affect image quality and reliability (11,12).

The major aim of this study was to compare the proportion of dental anomalies between the cleft side and non-cleft side in individuals with unilateral alveolar clefts (AC). Moreover, it will also be evaluated whether factors such as sex and side of AC (right or left) interfere with the proportion of dental anomalies on the non-cleft side.

## Material and Methods

-Sample

This was a descriptive and cross-sectional study of a series of 20 patients with non-syndromic unilateral AC registered at University Hospital of Aracaju, Aracaju, Brazil, using CBCT of the maxillary arch taken prior to alveolar bone grafting procedure. Exclusion criteria included bilateral or median AC; primary dentition; and history of orthodontic treatment or permanent teeth extraction based on the examination of the records. Ethnicity, sex, and side of cleft were not considered as they were believed to not interfere with the results. Ethical approval to conduct this study and written consent were obtained, and the principles outlined in the Declaration of Helsinki declaration, including its later amendments and revisions, were followed throughout.

-Image acquisition

Patients were positioned with the Frankfurt horizontal plane parallel to the floor. All CT scans were obtained by the same scanner i-CAT® Cone Beam 3-D Imaging System (Imaging Sciences International, Hatfield, PA, USA), from the occlusal plane to the nasal cavity. The technical parameters for image acquisition were 120 kV and 36 mA, and voxel size of 0.2 mm. The acquired images were stored in Digital Imaging and Communications in Medicine (DICOM) format. One single calibrated examiner converted the images into a 3D virtual model, by individually importing DICOM files to the DentalSlice Converter 2.1.5 (BioParts Prototipagem Biomédica, Brasília, DF, Brazil), a specific software for analysis and 3D reconstruction.

-3D image processing

**Figure 1:**
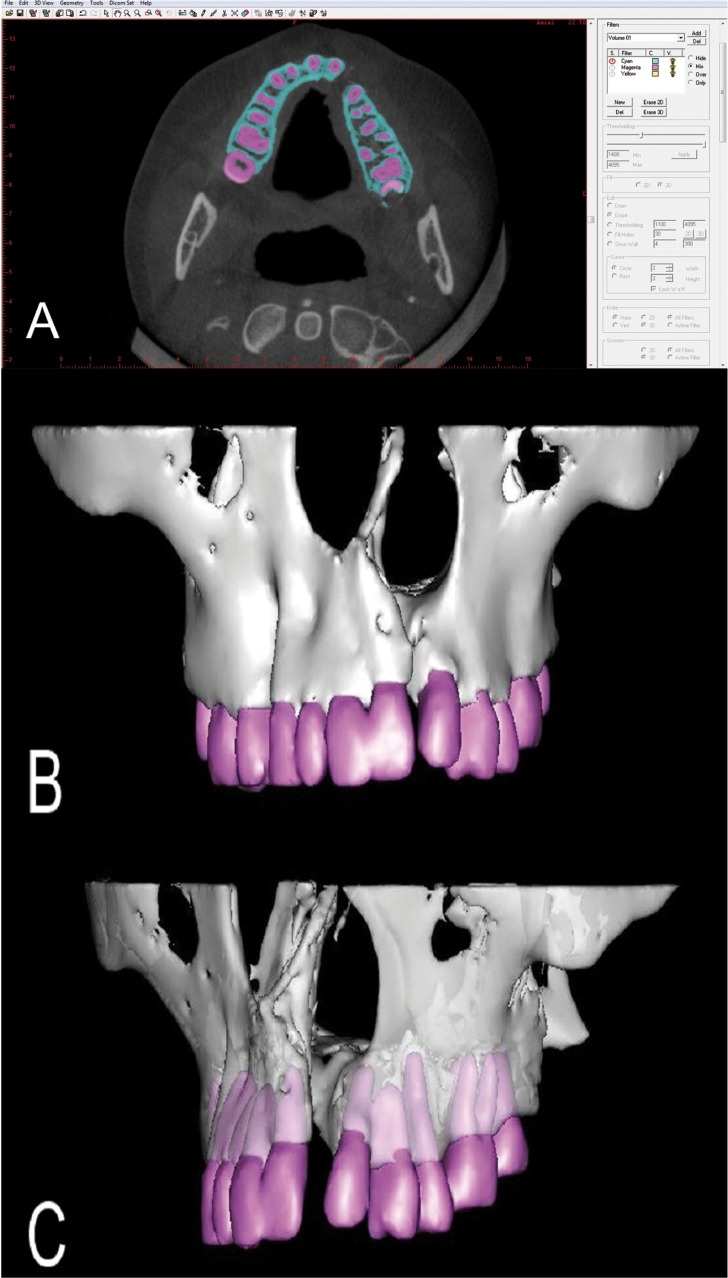
Screenshots from the 3D image processing: Segmentation of the structures of interest (A); 3D virtual model in a front view (B); 3D virtual model illustrating the features of rotation and transparency from a left view (C).

Once the DICOM file was imported, the slice thickness was reformatted and standardized at 0.75 mm. The threshold values for segmentation of the structures of interest (bone and teeth) were determined for each CBCT according to the density of tissues, and different colors were used (Fig. 1A).

After the manual segmentation was completed, the multiple slices were combined to create a 3D virtual model of the outlined structures (Fig. 1B). Different tools available in the software allowed the observer to freely rotate the reconstructed 3D object in all directions, transpire structures or isolate them completely, which favored the better visualization and interpretation of the image (Fig. 1C).

-Dental anomalies

To eliminate interexaminer differences, only one observer with experience in cleft treatment diagnosed the dental anomalies in all CT scans and 3D models. Data were recorded and subsequently evaluated.

Dental anomalies were classified as follows: (a) tooth agenesis - congenital absence of a tooth by developmental failure; (b) supernumerary tooth - a tooth adjacent to the AC additionally to the normal series, either mesially or distally when the lateral incisor is present; (c) giroversion - tooth rotation around its long axis; (d) microdontia - a tooth that is physically smaller than its contralateral homolog. The group of molars was not considered in this study.

-Statistical analysis

For descriptive analysis, mean and standard deviation values were used for continuous variables and absolute frequencies for discrete variables. For statistical analysis of paired data, McNemar’s exact test was used to compare the marginal frequencies, while Fisher’s exact test was used for unpaired data analysis. Statistical significance was determined at *p*<0.05. The results were evaluated with the aid of the statistical software RStudio 0.99.498 (RStudio, Inc., Boston, MA, USA) and Microsoft Excel 2010 (Microsoft Corporation, Redmond, WA, USA).

## Results

The subjects of the study consisted of 20 individuals, out of which, 11 (55%) were female and 9 (45%) were male. The mean age was 17.8±6.63 years. In 15 patients (75%) the AC was present on their left side.

 Table 1 shows the frequency of the various dental anomalies observed.

**Table 1:**
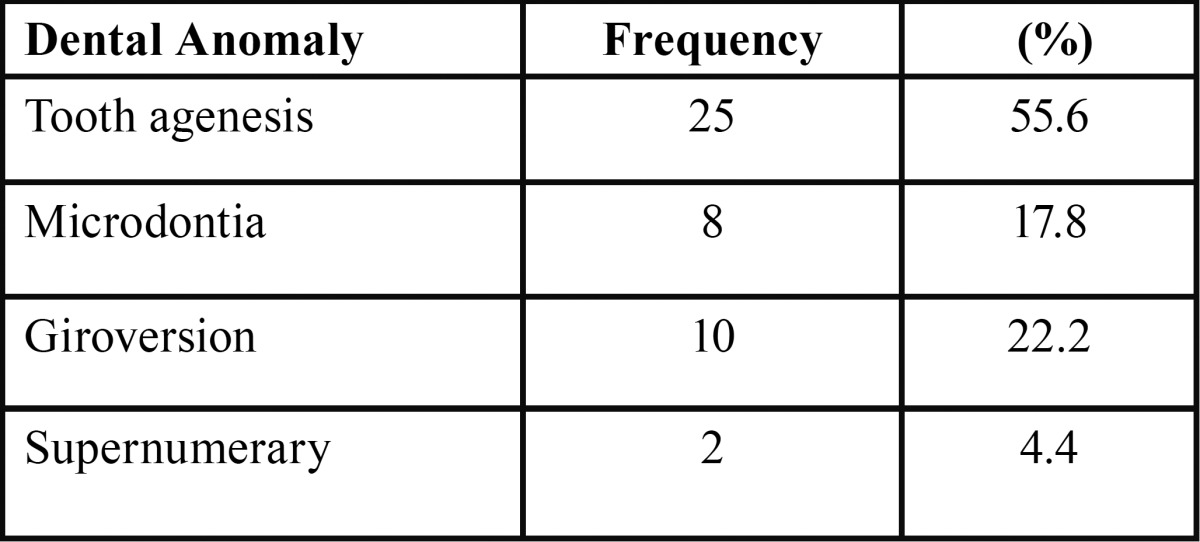
Frequency Of Occurrence Of The Various Dental Anomalies.

When compared the proportion of dental anomalies between cleft and non-cleft side of each individual, 8 patients (40%) had abnormalities on both sides, and 12 (60%) showed abnormalities only on the cleft side.

The prevalence of individuals with dental anomalies on the non-cleft side regarding the side of the cleft was also studied ( Table 2). Out of the 5 patients with AC on the right side, 3 (60%) showed abnormalities contralaterally; as for the 15 others with the AC located on the left side, dental anomalies were found on the right side in 5 (33.3%).

**Table 2:**
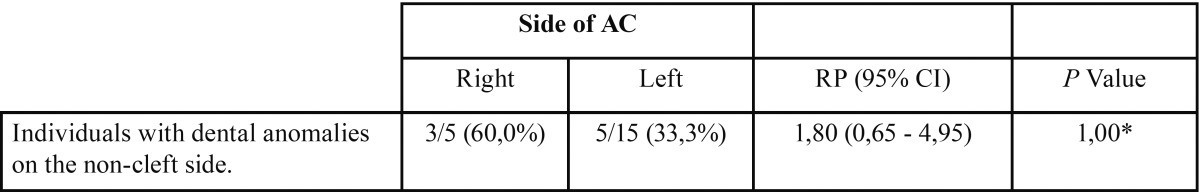
Prevalence Of Individuals With Dental Anomalies On The Non-Cleft Side According To The Sides Of Clefts.

Statistically significant differences were not found either between the prevalence of individuals with dental abnormalities on the non-cleft side and the sides of the AC (*p* = 1.00), or sex (*p* = 0.36) ( Table 2, Table 3).

**Table 3:**
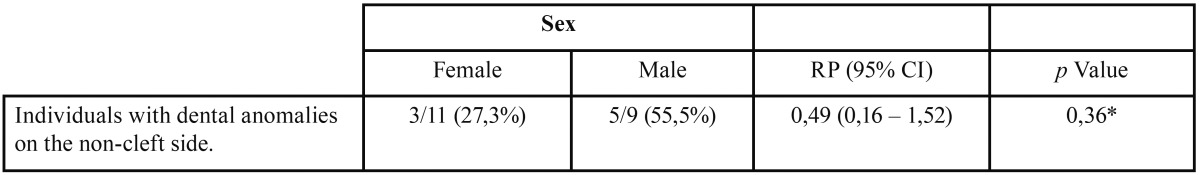
Prevalence Of Individuals With Dental Anomalies On The Non-Cleft Side In Relation To Sex.

Tooth agenesis was the most prevalent of the anomalies observed on cleft sides, affecting a total of 16 teeth. Accounting with 3 different types of anomalies, and 18 affected units - 9 of them affected by agenesis - the lateral incisor was the tooth most involved by dental anomalies. On the non-cleft sides, tooth agenesis was the only abnormality identified. In those cases, the second premolar was congenitally missing with a frequency twice higher than the lateral incisor, involving 6 and 3 teeth, respectively. A distribution of the relationship between the dental anomalies and teeth affected is described in  Table 4.

**Table 4:**
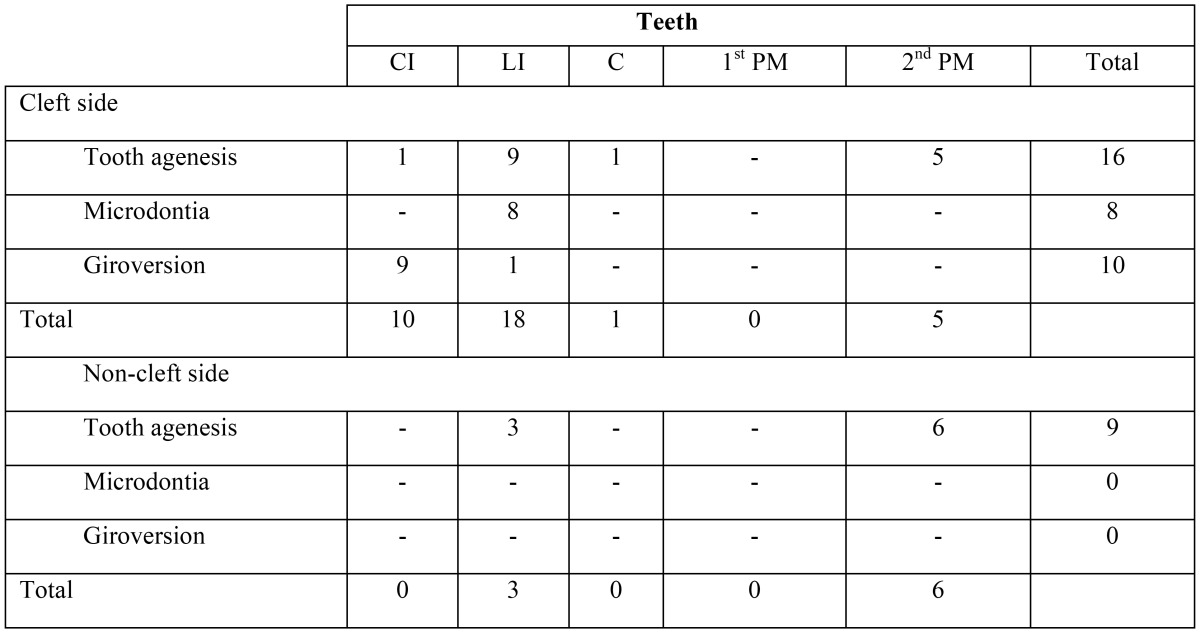
Descriptive Distribution Of The Dental Anomalies By Teeth Affected.

## Discussion

Individuals with oral clefts have higher frequency of dental anomalies when compared to the general population (7,13). The teeth most affected by agenesis was distributed in the following order: lateral incisors side on cleft side, second premolars on non-cleft side, second premolars on cleft side, and lateral incisors on non-cleft side.

The maxillary lateral incisor was the tooth most frequently affected by tooth agenesis, similarly to the study by Vanzin and Yamazaki(14).

Although it is not clear in the literature why lateral incisors adjacent to the AC are commonly involved by tooth agenesis, several authors have suggested different hypotheses. Tsai *et al.* (15), for instance, proposed that odontogenic potential of the lateral incisor comes from the maxillary and medial nasal prominences, therefore absence of fusion between those prominences due to deficiency of mesenchymal mass may result in agenesis of this tooth. Other authors mentioned that the cause for tooth agenesis in the cleft area could be justified by the deficiency of blood supply, either congenitally or secondary to surgery (16); or the bony defect itself caused by the cleft (17).

The second premolars were considerably affected by tooth agenesis, not only on the cleft side, but also on the opposite side, as reported by Ribeiro *et al.* (18) who showed that this group of teeth is the most affected outside the cleft region.

Our data showed that 40% of patients had abnormalities (tooth agenesis) in the second premolar and lateral incisor on the non-cleft side. In 2008, Menezes and Vieira (8) suggested the hypothesis that lateral incisors on the non-cleft side affected by dental anomalies could mean an unsuccessful bilateral clefts.

The giroversion was the second most frequent anomaly found (22.2%), primarily involving the central incisors adjacent to the AC. According to Smahel *et al.* (19), rotations of the central incisor may occur by insufficient space at the end of the alveolar segment to accommodate them.

The microdontia involved only a specific group of teeth: the lateral incisors next to the cleft area. Usually conical-shaped and typically shortened, such pattern of the tooth may be the result of the decreased growth potential of individuals with oral clefts (20), and possibly represent a partial expression of the same developmental failure which causes tooth agenesis (21). With a percentage of 17.8%, the microdontia was the third most frequent anomaly, resembling the results found by Sá *et al.* (22).

Supernumerary teeth had a frequency of 4.4%, close to the percentages of the studies by Sá *et al.* (22) and Kim and Baek (23), who found 3.4% and 5.4%, respectively.

As the frequency of dental anomalies on the non-cleft side showed no difference between the sexes (*p* = 0.36) or between the sides where the AC was located (*p* = 1.00); those findings may indicate that the reasons for anomalies outside the cleft region are not related to characteristics inherent to sex, but to a genetic background as proposed by Slayton *et al.* (24) who associated oral clefts with dental anomalies outside the cleft region to MSX1 and TGFβ3 genes. Other genes including TGFA, PAX9, FGFRI and IFR6, which had previously been related to oral clefts, were also associated with isolated tooth agenesis, as observed in premolars (25-27).

When the prevalence of abnormalities on non-cleft side was compared to the side where the cleft was located, it was observed that 60% of patients with right AC showed abnormalities on the left side. Although that finding was not statistically significant, due to the high percentage obtained, we suggest further studies to test the hypothesis with a larger sample that the side of the AC (right or left) may have influence upon the prevalence of dental anomalies on the opposite side.

It is important to note that in this study the CBCT reconstructed in 3D virtual models replaced the conventional panoramic radiographs to diagnose the dental anomalies, providing high-quality images on the morphology and position of teeth observable from different angles, as well as free of overlaps and distortions. Nevertheless, since CBCT is a very costly procedure, the cost-effectiveness in terms of detection and the contribution towards the benefit in the management of the cleft must be clearly defined. In this research, the authors employed CBCT scans which were solicited by oral and maxillofacial surgeons for cleft analysis prior to bone grafting procedure.

Conclusions

Aware of the predisposition of cleft individuals to present dental anomalies and consequently the impact on maxillofacial anatomy, speech, appearance and masticatory functions, the team involved in their rehabilitation must carefully consider such disturbances during treatment planning as part of a comprehensive cleft care.

The use of CBCT 3D virtual models to assess dental anomalies in a group of subjects with non-syndromic unilateral AC allowed precision and improvement in diagnosis. Based on the results presented, our study showed a high frequency of dental anomalies in the cleft individuals, as well as indicated that the side of AC and sex do not interfere in the proportion of dental anomalies on non-cleft side.

Furthermore, once the second premolar on the non-cleft sides were the teeth primarily affected by agenesis, future research should consider investigating in a larger sample whether the side of AC has influence on the proportion of dental anomalies on the non-cleft side as well as its possible etiological reasons.
